# Postprandial plasma amino acid and appetite responses to a low protein breakfast supplemented with whey or pea protein in middle-to-older aged adults

**DOI:** 10.1007/s00394-025-03605-0

**Published:** 2025-02-11

**Authors:** Marie Korzepa, Ryan N. Marshall, Lucy M. Rogers, Archie E. Belfield, Jonathan I. Quinlan, Yijia Huang, Ari Gritsas, Tyler A. Churchward-Venne, Elisa I. Glover, Luc J. C. van Loon, Gareth A. Wallis, Leigh Breen

**Affiliations:** 1 School of Sport, Exercise and Rehabilitation Sciences, University of Birmingham, Birmingham, UK; 2 Department of Kinesiology and Physical Education, McGill University, Montreal, QC Canada; 3 Research Institute of the McGill University Health Centre, Montreal, QC Canada; 4 Division of Geriatric Medicine, McGill University, Montreal, QC Canada; 5 Volac International LTD, Royston, Hertfordshire UK; 6 Department of Human Biology, NUTRIM, Maastricht University Medical Centre+, Maastricht, The Netherlands; 7 NIHR Birmingham Biomedical Research Centre, University Hospitals Birmingham NHS Foundation Trust, Birmingham, UK

**Keywords:** Ageing, Dietary protein, Protein quality

## Abstract

The addition of low-dose protein to low protein-containing meals in middle-to-older aged adults may promote greater postprandial plasma aminoacidemia and mitigate declines in muscle health but may be dependent on the source and quality of protein consumed. This single-blind randomised study investigated postprandial plasma aminoacidemia and appetite regulatory responses to a typical lower protein-containing (∼0.07 g·kg body mass[BM]^−1^) mixed breakfast supplemented with ∼0.13 g·kg BM^− 1^ of whey protein concentrate (MB + WPC) or pea protein isolate (MB + PPI) in middle-to-older aged adults. Venous blood samples were collected whilst fasted and over a 180-min postprandial period to determine plasma amino acid and appetite hormone concentrations. Perceived appetite ratings were measured using Visual Analogue Scales. Plasma total amino acids and essential amino acids increased over time (both *P* < 0.05) with no between-group differences or overall availability over 180 min (incremental area under the curve (iAUC)). Plasma leucine concentrations increased over time (both *P* < 0.05) with greater peak concentrations (*P* = 0.032) and iAUC (*P* = 0.012) in MB + WPC compared with MB + PPI. Plasma total-ghrelin and total-GLP-1 concentrations and perceived ratings of hunger, fullness, and satiety were transiently altered following MB + WPC and MB + PPI (*P* < 0.05 for all), with no differences between groups. In middle-to-older aged adults, co-ingesting a small bolus of whey protein alongside a typical lower protein-containing breakfast elicits greater plasma leucinemia than co-ingestion of pea protein, with no differential impact on appetite regulation. Whether the addition of low-dose whey protein to typical meals of middle-to-older aged adults can enhance muscle anabolism without adversely affecting appetite remains to be determined.

**Supplementary Information:**

The online version contains supplementary material available at 10.1007/s00394-025-03605-0.

## Introduction

Age-related skeletal muscle mass and strength loss, termed ‘sarcopenia’ [1], is associated with numerous comorbidities, loss of independence and high socio-economic burden. Sarcopenia progression is underpinned by a diminished muscle protein synthesis (MPS) response to dietary protein ingestion [2, 3], particularly at low-to-moderate protein doses [4–6]. It is suggested that dysregulation of MPS [7, 8] and the onset of sarcopenia commences in middle age [9]. Considering the increasing prevalence of sarcopenia [10] the development of physical activity and dietary intervention strategies from middle age onwards may be preferred to preserve muscle health with advancing age.

Compared with younger adults, older adults are recommended to consume more protein with each meal to maximally stimulate MPS [4, 11, 12]. This notion is reinforced by evidence linking higher daily dietary protein intake with superior muscle health outcomes in older adults [13]. However, increasing dietary protein intake in older adults is impeded by reduced appetite [14, 15], taste and sensory impairments [16], digestive issues [17], and psychosocial factors [18, 19]. Henceforth, protein intakes which maximise MPS are not commonly achieved in community-dwelling middle-to-older age adults and are further compromised during hospitalisation [20]. Achieving daily protein requirements is further perturbed by uneven protein distribution throughout the day [21, 22]. For example, typical protein intake at breakfast is below the threshold required for robust MPS stimulation [21, 23], theoretically prolonging overnight negative net muscle protein balance [24]. Conversely, higher protein-containing breakfasts are conducive to greater daily protein intake [25] and superior muscle health outcomes in older adults [11, 26].

Given the difficulties older adults have in consuming sufficient protein for PS stimulation, the source and quality of ingested protein may modulate postprandial muscle anabolism in this population [27]. Dietary protein quality is determined by the constituent amino acid (AA) profile, particularly the essential amino acids (EAAs) and leucine, and overall digestibility [28]. Compared to animal-based sources, most plant-based protein sources are generally lower in digestibility and deficient in at least one EAA [29]. Indeed, postprandial MPS stimulation is generally greater with animal-derived compared with plant-derived proteins, when consumed in isolated supplemental form [30, 31] or in a mixed meal [32]. Importantly, many traditional breakfasts contain more lower quality plant-based proteins (i.e., cereals, breads, fruit) than other meals [33, 34]. Given the typically low quality and amount of ingested protein at breakfast, supplementation to bolster EAAs/leucine may benefit MPS potential in middle-to-older aged adults [35]. Given postprandial aminoacidemia may influence hunger [36–38], the appetite response following a protein-supplemented breakfast requires consideration to ensure prolonged satiety is not observed (typical of high-protein meals [39, 40]) which may compromise subsequent feeding opportunities.

The present study aimed to investigate postprandial plasma aminoacidemia and indices of appetite regulation following the ingestion of low protein-containing whole food mixed breakfast, supplemented with a small bolus of whey protein concentrate or pea protein isolate in middle-to-older aged adults. We hypothesised that a small bolus of whey protein concentrate to a lower protein-containing breakfast would elicit a more pronounced postprandial EAA and leucine response than an isonitrogenous amount of pea protein isolate. Furthermore, we posited that appetite regulation and associated hormonal mechanisms would be transiently yet similarly altered following breakfast consumption and whey or pea protein co-ingestion.

## Methods

### Participants

Twenty-seven active and otherwise healthy males and females aged 50–70 y old volunteered to participate in a single-blind parallel-designed study. Participants were allocated to a predefined participant code according to sex, with condition randomised before study onset using RAND function in Excel (Microsoft, USA) to assign groups. Groups consumed a low-protein containing mixed breakfast supplemented with either a small bolus of whey protein concentrate (MB + WPC) or pea protein isolate (MB + PPI). Complete plasma samples were collected for 22 participants (MB + WPC; *n* = 13, MB + PPI; *n* = 9), the characteristics for whom are presented in Table 1. Ethical approval was obtained by the NHS Surrey Borders Research Ethics Committee (21/LO/0401) with all procedures conducted in accordance with the Declaration of Helsinki. The present study was the first meal of a 10-day intervention as part of a larger experimental trial, which was prospectively registered at ClinicalTrials.gov (NCT05574205) which subsequently restricted the present study to being single-blind. Participant flow through the study protocol is outlined in a CONSORT diagram in Supplemental Fig. 1.

**Table 1:** Participant characteristics

	MB + WPC (*n* = 13)	MB + PPI (*n* = 9)	Pooled (*n* = 22)	*P value*
Male/Female	6/7	5/4	11/11	
Age (y)	64.2 ± 4.4	57.1 ± 7.0	61.3 ± 6.5	0.008*
Height (m)	1.69 ± 0.10	1.71 ± 0.13	1.70 ± 0.11	0.769
Mass (Kg)	71.6 ± 9.8	74.4 ± 20.3	72.7 ± 14.7	0.668
BMI (Kg/m^2^)	24.9 ± 1.8	25.1 ± 3.7	25.0 ± 2.7	0.866
Body fat (%)	26.1 ± 7.1	28.2 ± 8.8	27.0 ± 7.7	0.192
RER	0.89 ± 0.1	0.81 ± 0.1	0.86 ± 0.1	0.998
RMR (kcal)	1474 ± 337	1621 ± 618	1576 ± 479	0.456
Sedentary time (%)	72.2 ± 11.2	65.6 ± 13.3	70.0 ± 12.3	0.250
MVPA (%)	17.7 ± 7.3	24.1 ± 8.1	20.5 ± 0.7.7	0.082

BMI: body mass Index; MB + PPI; mixed breakfast with pea protein isolate; MB + WPC: mixed breakfast with whey protein concentrate; MVPA: moderate and vigorous physical activity; RER: respiratory exchange ratio; RMR: resting metabolic rate. Data presented as mean ± SD. Independent samples T-test identified any significant differences between pea protein isolate and whey protein concentrate. * Denotes significant differences between groups (*P* < 0.05)

### Preliminary assessments

Participants were screened for eligibility before providing written informed consent to participate. Exclusion criteria were: BMI > 30 kg⋅m^2^, any underlying metabolic health conditions or dietary intolerances which may cause allergic reactions. Anthropometrics were collected for height using a stadiometer (SECA, Hamburg, Germany) and body mass by digital scale (OHAUS, Champ II, Switzerland). Following preliminary testing, participants kept a diet diary where all items were weighed for at least 3 days prior to the intervention (including 1 weekend day) and were provided with a wrist-worn accelerometer (GENEActiv, ActivInsights, UK) to characterise habitual dietary intake and physical activity.

### Experimental testing

**Fig. 1:**
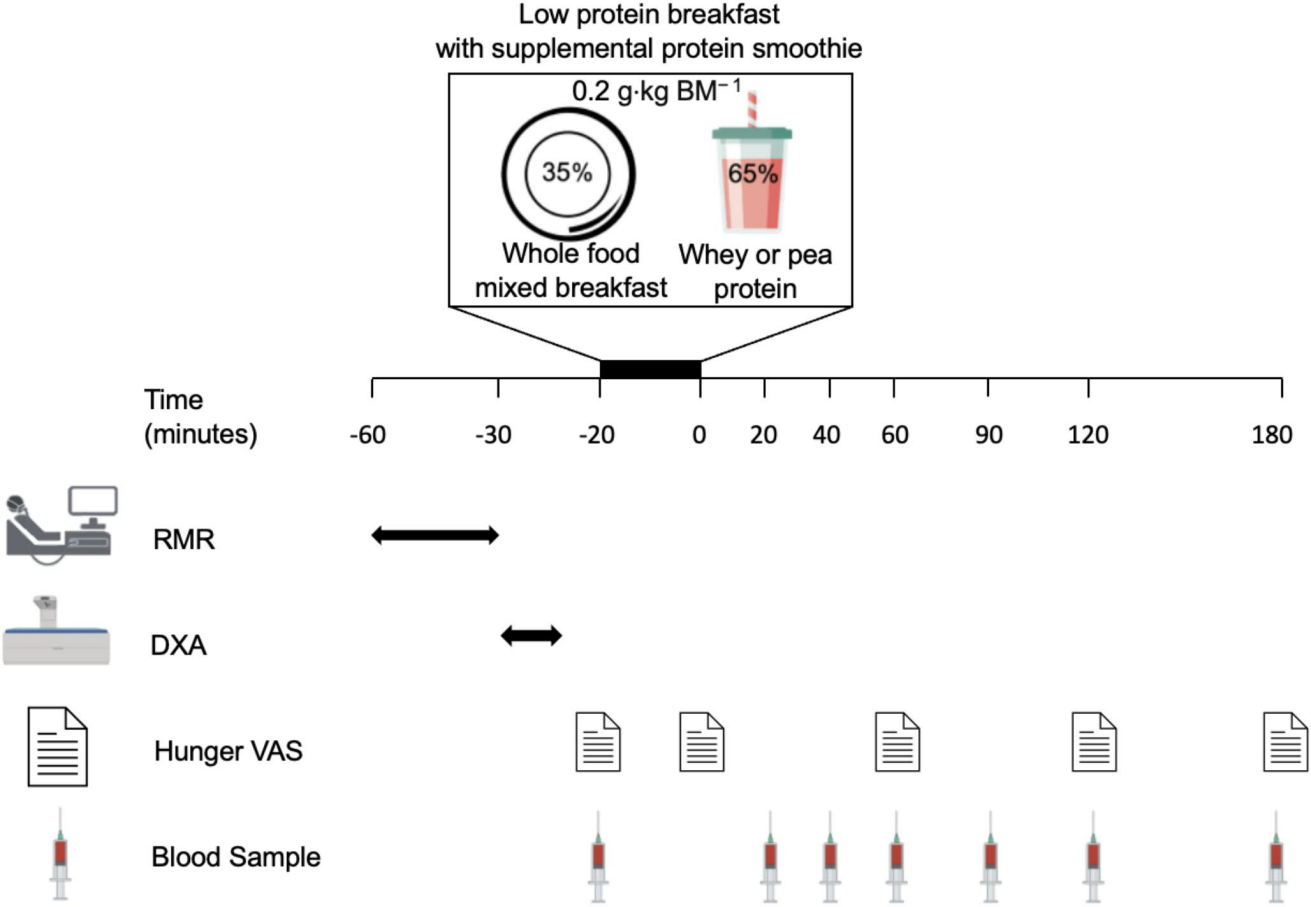
Study Schematic. BM: body mass; DXA: Dual-energy x-ray absorptiometry; RMR: Resting Metabolic Rate; VAS: Visual Analogue Scale

A schematic overview of the experimental period can be seen in Fig. 1. Participants arrived at the University of Birmingham, School of Sport, Exercise and Rehabilitation Sciences having fasted for > 10 h and abstained from strenuous exercise 24 h prior. Anthropometrics were reassessed to confirm initial measurements made during preliminary testing. Whilst fasted, body composition was measured via dual-energy x-ray absorptiometry (DXA; Discovery A, Hologic, Bedford, MA). Resting metabolic rate (RMR; CPX Jaeger, Vyntus) was also evaluated with the participant laying supine whilst wearing a Hans Rudolf face mask (Cranlea, Birmingham, UK) during 20 min to collect RMR and respiratory exchange ratio (RER), as described previously [41]. Thereafter, a cannula (BD Venflon™, USA) was inserted antegrade into an antecubital forearm vein to obtain a fasted blood sample (t=-20 min). The cannula device was flushed with 5 mL sterile NaCl 0.9% (BD PosiFlushTM, USA) after each collection to maintain patency. Participants then consumed a whole-food mixed breakfast alongside a small bolus of supplemental whey or pea protein smoothie. Following breakfast consumption, further blood samples were obtained at 0, 20, 40, 60, 90, 120 and 180 min of the postprandial phase. A nine-question Visual Analogue Scale (VAS; Flint et al., 2000) [42] was used to measure perceived appetite, hunger, and taste at t= -20, 0, 60, 120 and 180 min. Participants were instructed to draw a vertical line through a horizontal 0–100 mm VAS for 9 questions at each measurement time-point, with 0 being the least and 100 being the most for thirst, hunger, satisfaction and fullness and 0 being very much and 100 being not at all for taste preference for something sweet, salty, savoury, A composite appetite score was calculated using the first 4 questions on the VAS sheet, as previously described [43]. For statistical analysis, all VAS-derived measures, including appetite composite score, were expressed as change from baseline (t= -20 min).

### Whole-food breakfast and protein supplements

The lower protein-containing whole-foods breakfast provided 0.07 g⋅kg body mass[BM]^− 1^ of protein for both groups (MB + WPC; 6.5 ± 0.6 g, MB + PPI; 6.8 ± 1.0 g, *P* = 0.364). Foods consumed for the breakfast test meal were identical for each condition (muesli, yoghurt and blueberries), in which 66–80% of protein was provided from plant-derived sources, which is comparable to typical omnivorous breakfasts consumed by community-dwelling older adults [44]. Alongside the breakfast meal, participants consumed a small bolus (< 150 mL) protein smoothie beverage containing 0.13 g⋅kg BM^− 1^ of protein from whey protein concentrate (WPC; Volactive^®^ UltraWhey Sugar Free WPC, Volac, Hertfordshire, UK) or pea protein isolate (PPI; MyProtein™, The Hut Group, Manchester, UK). The 0.13 g·kg BM^− 1^ equated to 7.89 ± 1.55 g (range: 5.62–11.65 g) of protein for both treatments, or 8.00 ± 1.10 g (range: 6.39–9.73 g) and 7.74 ± 2.11 g (range: 5.62–11.65 g) of WPC and PPI powder, respectively. Total breakfast *plus* supplemental smoothie protein intake was 15.88 ± 2.67 g (range: 12.50–23.20 g) of protein for both groups (MB + WPC; 15.4 ± 1.6 g, MB + PPI; 16.5 ± 3.7 g). The constituent elements of the smoothie were identical for each condition, apart from the supplemental powder used. The details of the AA profile of supplements used are provided in Table 2 with the macronutrient and energy of the WPC and PPI-supplemented breakfasts provided in Table 3. The details of the protein smoothie recipe are provided in Supplemental Fig. 2.

**Table 2:** Amino acid content of protein supplements

Content per 100 g of powder (g)	Whey Protein Concentrate (Volactive Sugar Free)	Pea Protein Isolate (MyProtein)
Aspartic Acid	9.15	8.99
Serine	4.50	4.18
Glutamic acid	14.50	12.5
Glycine	1.65	3.13
Histidine	1.45	1.78
Arginine	2.00	6.12
Threonine	5.70	2.69
Alanine	4.20	3.17
Proline	5.00	3.46
Cystine	2.00	0.67
Tyrosine	2.00	2.77
Valine	4.00	3.04
Methionine	2.00	0.80
Lysine	8.10	5.90
Isoleucine	4.50	2.57
Leucine	8.10	5.75
Phenylalanine	2.50	3.74
Tryptophan	1.40	-
**∑ Determined amino acids**	**82.75**	**71.26**
**∑ EAA**	**37.75**	**26.27**
**∑ NEAA**	**45.00**	**44.99**

EAA: essential amino acids; NEAA: non-essential amino acids

**Table 3:** Energy and macronutrient content of mixed breakfast test meals

	MB + WPC	MB + PPI	*P* value
Carbohydrate (g)	35.8 ± 5.3	38.4 ± 5.7	0.295
Carbohydrate (g·kg BM^− 1^)	0.51 ± 0.05	0.53 ± 0.08	0.276
Fat (g)	7.65 ± 0.71	6.87 ± 1.27	0.080
Fat (g·kg BM^− 1^)	0.11 ± 0.01	0.09 ± 0.01	0.013*
Protein (g)	15.80 ± 1.98	16.56 ± 3.67	0.092
Protein (g·kg BM^− 1^)	0.22 ± 0.01	0.23 ± 0.01	0.538
Supplemental protein (g)	8.00 ± 1.10	7.74 ± 2.11	0.705
Supplemental protein (g·kg BM^− 1^)	0.13 ± 0.0	0.13 ± 0.0	0.990
Proportion animal protein (%)	70.94 ± 2.09	10.07 ± 1.28	< 0.001**
Fibre (g)	5.24 ± 0.66	5.85 ± 0.83	0.071
Fibre (g·kg BM^− 1^)	0.07 ± 0.01	0.08 ± 0.01	0.070
Total energy (Kcal)	303 ± 34	311 ± 49	0.683

* denotes significant difference between conditions (*P < 0.05, **P < 0.001)

### Plasma analysis

Blood samples were collected in EDTA-coated tubes (BD Vacutainer^®^, USA). Samples were placed on ice until the end of the collection and then centrifuged at 3500*g* for 15 min at 4°C with collected plasma stored in -80 °C freezers until data collection was completed for all participants. Plasma samples were aliquoted out separately for later plasma glucose determination, AA concentration and appetite hormone analysis to minimise repeated freeze-thaw cycles of samples.

Plasma glucose was determined on the automated Daytona RX + using the glucose hexokinase kit (Randox Laboratories, UK) with all samples run in duplicate. Enzyme-linked immunosorbent assay (ELISA) kits were used to measure plasma-derived insulin (Mercodia, Uppsala, Sweden), total GLP-1 and total ghrelin (Merck Millipore, Darmstadft, Germany), with all samples run in duplicate and within participant samples run on the same plate for timepoints t= -20, 20, 40, 60, 90, 120 and 180 min.

AA concentrations within plasma were analysed using reversed-phase ultra-performance liquid chromatography-mass spectrometry (UPLC-MS) on an Agilent 6460 triple quadrupole mass spectrometer coupled with an Agilent 1290 UPLC system (Agilent CA, USA) using methods in Supplementary Material 1, adapted from [37]. Plasma AA data are presented in µmol⋅L^− 1^.

### Statistical analysis

A sample size of *n* = 9 per group with power(1-β) = 0.95 was calculated using G*Power (Ver 3.1, Heinrich Hein University) derived from differences in plasma leucine availability between vegan and omnivorous whole-food meals in older adults from Pinckaers et al. [32]. Participant characteristics and AA comparison tables were created in Excel (Microsoft, USA). Figures were produced using GraphPad Prism with statistical significance set a priori as *P* ≤ 0.05. For all time-dependant and time-independent variables of plasma and VAS-derived outcomes over the 180 min postprandial period, a two-way repeated measures analysis of variance (ANOVA) was completed. In the instance of missing timepoints, a mixed-effect model was performed. The fasted baseline value was used as the reference point for statistical comparisons when running time-course ANOVAs. Incremental area under the curve (iAUC) and where appropriate, total area under the curve (AUC) for postprandial metabolite, appetite hormone and VAS responses were calculated with the trapezoid method using the Time Series Response Analyser [45]. In instances where there were statistically significant interaction effects (time x group), a Bonferroni post-hoc test was run to locate statistical differences between specific parameters. Data are presented as mean ± standard error of the mean (SEM) unless otherwise stated.

## Results

### Participant characteristics

Participant characteristics are presented in Table 1. The mean age of participants was significantly lower for MB + PPI compared with WPC (*P* = 0.008). There were no differences in sedentary or moderate and vigorous activity (MVPA) time, RER, RMR, and body composition measures between groups.

### Plasma aminoacidemia

**Fig. 2:**
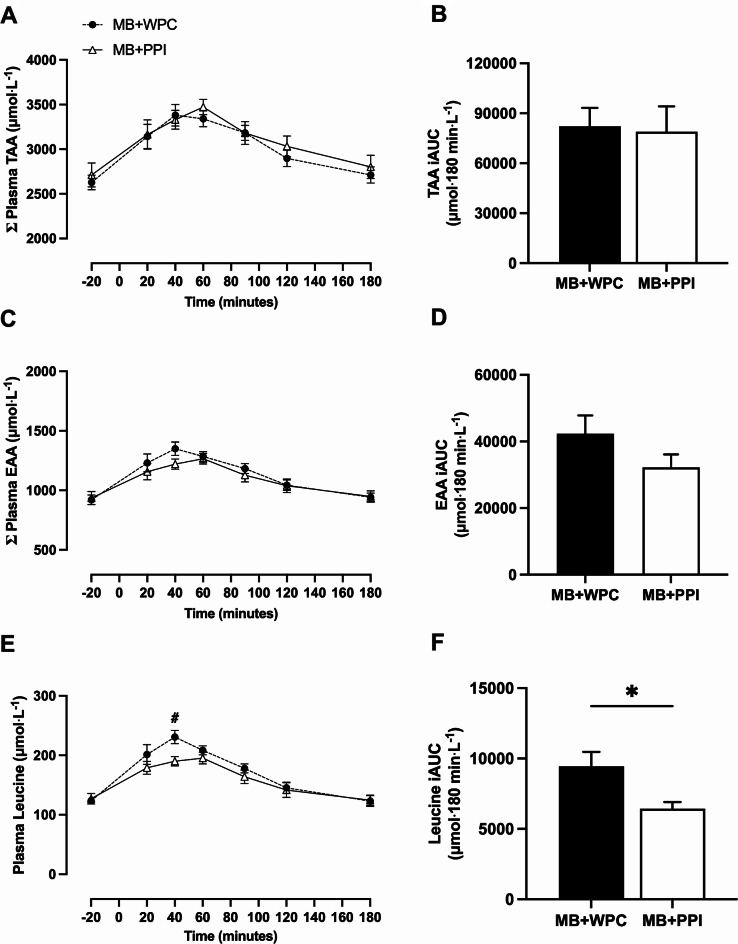
Plasma total amino acid (TAA), essential amino acid (EAA) and leucine concentrations. Postprandial plasma TAA (**A**) and incremental area under the curve (iAUC) (**B**) over 180 min. Postprandial EAA excursions (**C**) and corresponding iAUC over 180 min (**D**). Plasma leucine excursions (**E**) and iAUC response (**F**). Data are presented as mean and SEM with *n* = 13 for mixed breakfast with whey protein concentrate (MB + WPC) and *n* = 9 for mixed breakfast with pea protein isolate (MB + PPI). Two-way ANOVA showed a significant effect of time for **A**, **C** and **E** (*P* < 0.001) but no group or clear interaction effect, apart from close interaction for **E**(*P* = 0.057). Independent samples t-test was used to compare between groups iAUC for TAA (**B**), EAA, (**D**) and leucine (**F**). *Denotes significant difference (*P* < 0.05) and # denotes significant difference between groups at indicated timepoint due to close interaction effect in **E**

Plasma total amino acid (TAA) concentrations significantly increased over time from basal (t=-20 min) (Fig. 2A; *P* < 0.001) with no significant main effect of group (*P* = 0.247), or group x time interaction (*P* = 0.915). There were no between-group differences in peak plasma TAA concentrations (*P* = 0.517) or time-to-peak (*P* = 0.377). The plasma TAA iAUC was not significantly different between groups (Fig. 2B; *P* = 0.865).

Plasma essential amino acid (EAA) concentrations significantly increased over time (Fig. 2C; *P* < 0.001) with no significant main effect of group (*P* = 0.531), or group and time interaction (*P* = 0.368). There were no between-group differences in peak plasma EAA concentrations (*P* = 0.366) or time-to-peak (*P* = 0.757). The plasma EAA iAUC was not significantly different between groups (Fig. 2D; *P* = 0.190).

Plasma leucine concentrations significantly increased over time (Fig. 2E; *P* < 0.001), with no significant main effect of group (*P* = 0.258) or group and time interaction (*P* = 0.057). Considering the trend for group and time interaction, it was noted that plasma leucine concentrations were significantly greater with MB + WPC compared with MB + PPI at 40 min into the postprandial phase (WPC: 230 ± 40 µmol⋅L^− 1^; PPI: 190 ± 23 µmol⋅L^− 1^, *P* = 0.006). There were no between-group differences in peak plasma leucine concentrations (*P* = 0.081) or time-to-peak (*P* = 0.784). The plasma leucine iAUC was greater in MB + WPC compared with MB + PPI (Fig. 2F; *P* = 0.032).

The remaining plasma AA concentrations and the corresponding iAUC are presented in Supplemental Figs. 4 and 5. Briefly, postprandial plasma amino acid concentration significantly increased over time from fasted values for all AAs with the exception of cystine and glycine (both *P* > 0.05). During the 180 min timecourse response, two-way ANOVA revealed time x group interaction effect for plasma arginine (*P* = 0.012) and isoleucine (*P* = 0.044), with main group differences for plasma phenylalanine (*P* < 0.001) and tyrosine (*P* = 0.008) (Supplemental Fig. 4). Bonferroni post hoc analysis unveiled that plasma arginine concentration was significantly (*P* > 0.05) greater in MB + PPI than MB + WPC at t = 60 and t = 120, whereas plasma isoleucine concentrations were significantly greater in MB + WPC than MB + PPI at t = 40 (Supplemental Fig. 4). Peak plasma phenylalanine (*P* < 0.001) and asparagine (*P* = 0.048) concentrations were greater in MB + PPI than MB + WPC, whereas peak plasma aspartic acid (*P* = 0.038) concentration was greater in MB + WPC than MB + PPI. The iAUC for plasma arginine (*P* = 0.004), glycine (*P* = 0.022), methionine (*P* = 0.026) and phenylalanine (*P* = 0.018) was significantly different between groups (Supplemental Fig. 5).

### Plasma glucose and insulin

**Fig. 3:**
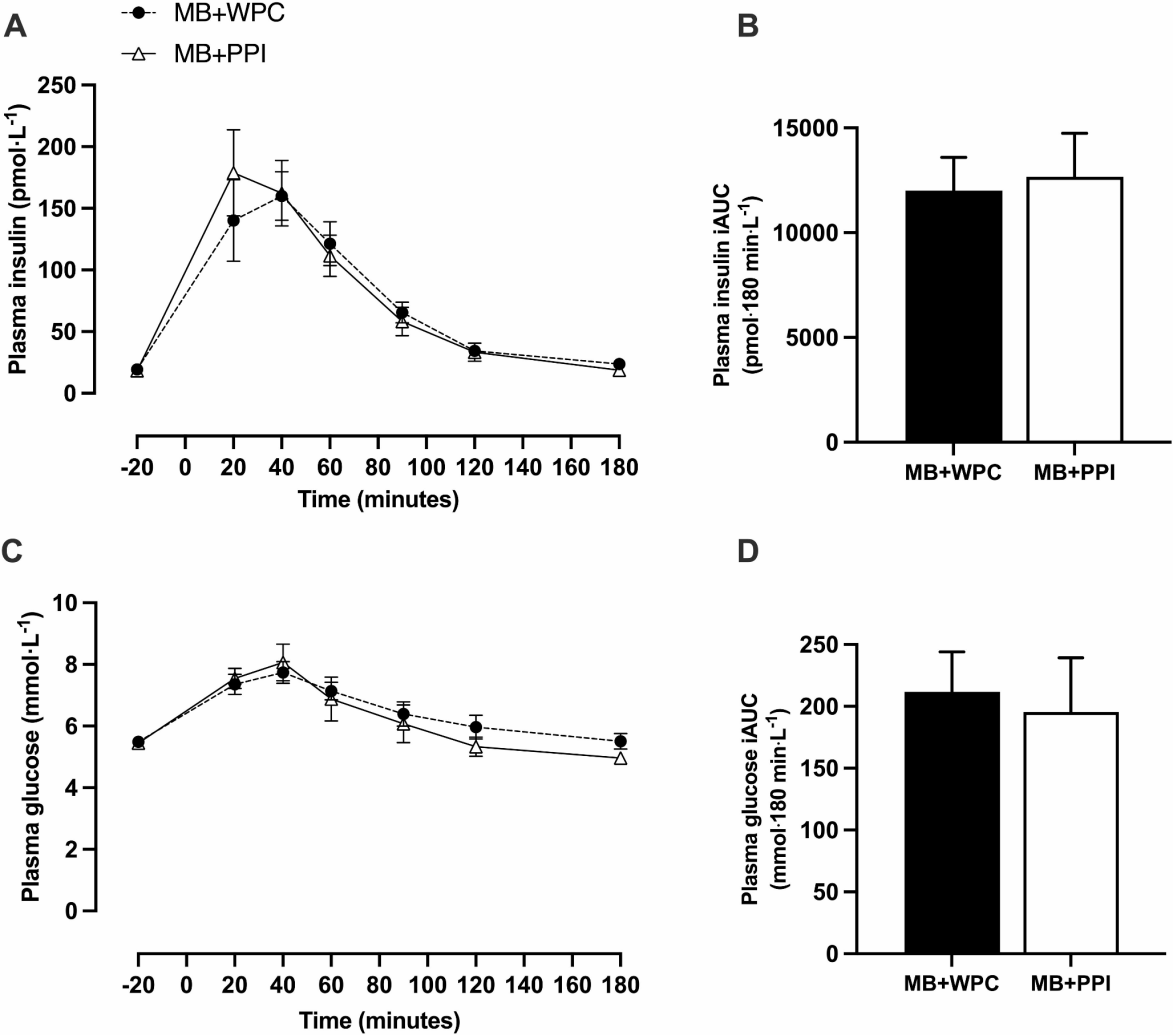
Insulin and Glucose excursions and area under the curve. Postprandial plasma insulin excursions (**A**) and incremental area under the curve (iAUC) (**B**) over 180 min and postprandial glucose excursions (**C**) and iAUC over 180 min (**D**). Data are presented at mean and SEM with *n* = 13 for mixed breakfast with whey protein concentrate (WPC) and *n* = 9 for mixed breakfast with pea protein isolate (PPI). 2-way ANOVA was run to test for significant difference between groups and timepoints, with baseline value (t=-20) within condition set as the reference point. Independent samples t-test was used to compare insulin iAUC (**B**) and Glucose iAUC (**D**). Denotes significant difference from baseline for both groups (*P* < 0.05)

Significant increases over time were seen for plasma insulin (Fig. 3A; *P* < 0.001) and plasma glucose (Fig. 3C; *P* < 0.001) with no significant main effect of group for insulin (*P* = 0.844) and glucose (*P* = 0.650) or group and time interaction for plasma insulin (*P* = 0.791) and plasma glucose (*P* = 0.483). There were no between-group differences in peak plasma insulin (*P* = 0.203) and plasma glucose concentrations (*P* = 0.109) or time-to-peak plasma insulin (*P* = 0.980) and plasma glucose (*P =* 0.946). There was no significant difference between groups in iAUC for plasma insulin (Fig. 3B, *P* = 0.797) or plasma glucose (Fig. 3D, *P* = 0.764).

### Plasma appetite hormones

**Fig. 4:**
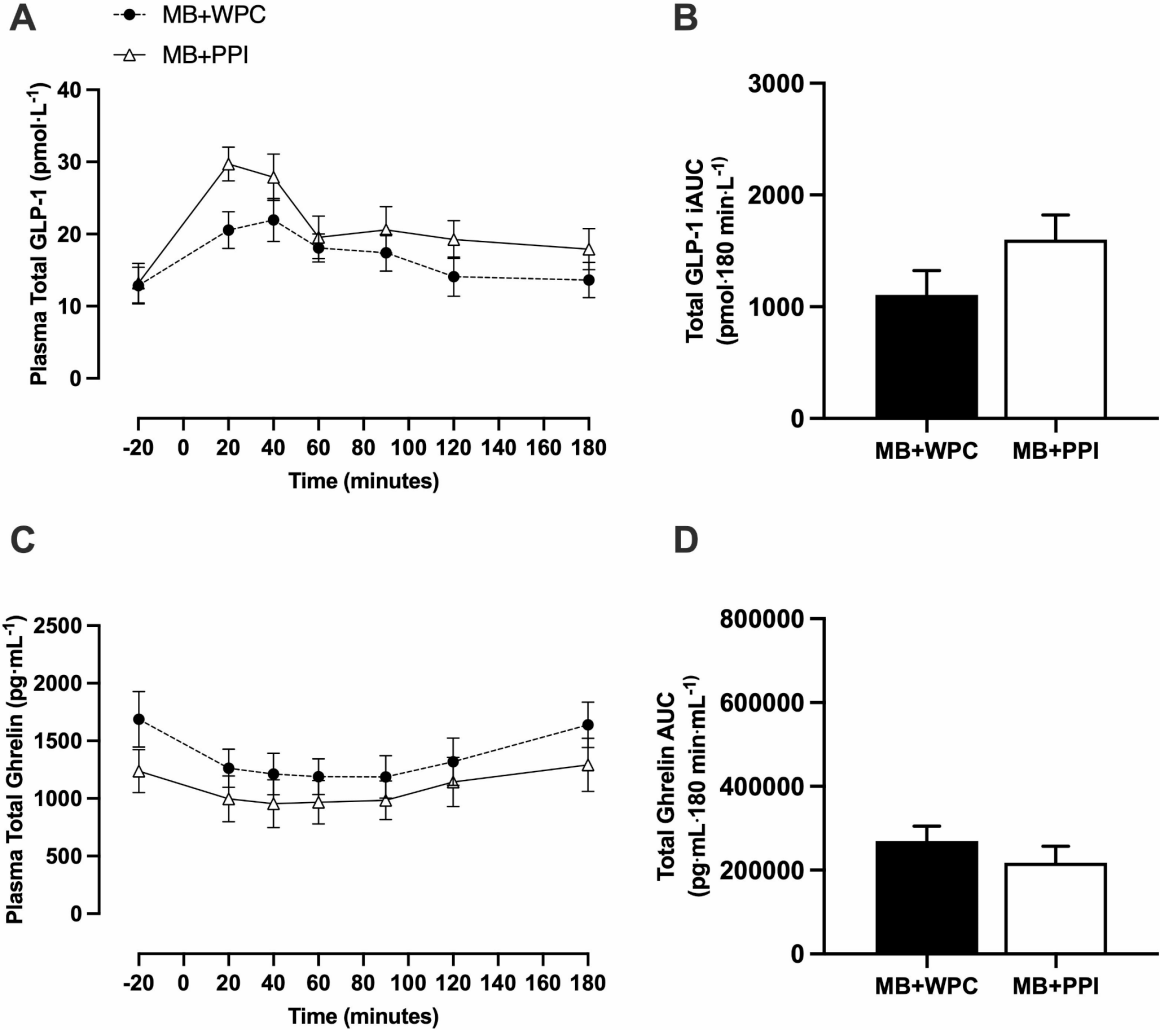
Postprandial appetite hormone response following pea or whey protein supplemented mixed breakfast. Postprandial total GLP-1 excursions (**A**) and incremental area under the curve (iAUC) (**B**) over 180 min and postprandial total ghrelin excursions (**C**) and total area under the curve (AUC) over 180 min (**D**). Data are presented at mean and SEM with *n* = 13 for mixed breakfast with whey protein concentrate (MB + WPC) and *n* = 9 for mixed breakfast with pea protein isolate (MB + PPI). 2-way ANOVA was run to test for significant differences between groups and timepoints and overall interaction with baseline value (t=-20) within condition set as the reference point for **A** and **C**. Independent samples t-test was used to compare total GLP-1 iAUC (**B**) and total Ghrelin AUC (**D**)

Plasma total GLP-1 concentrations were significantly increased with time compared to baseline (Fig. 4A; *P* < 0.001) with no significant main effect of group (*P* = 0.212), or group and time interaction (*P* = 0.131). There were no between-group differences in peak plasma total GLP-1 concentrations (*P* = 0.187) or time-to-peak (*P* = 0.533). The plasma total GLP-1 iAUC was not significantly different between groups (Fig. 4B; *P* = 0.134). Plasma total ghrelin concentrations significantly decreased over time (Fig. 4C; *P* < 0.001) with no significant main effect of group (*P* = 0.316), or group and time interaction (*P* = 0.592). There were no between-group differences in nadir plasma total ghrelin concentrations (*P* = 0.496) or time-to-nadir (*P* = 0.561). The plasma total ghrelin AUC was not significantly different between groups (Fig. 4D; *P* = 0.345).

### Perceived appetite responses

**Fig. 5:**
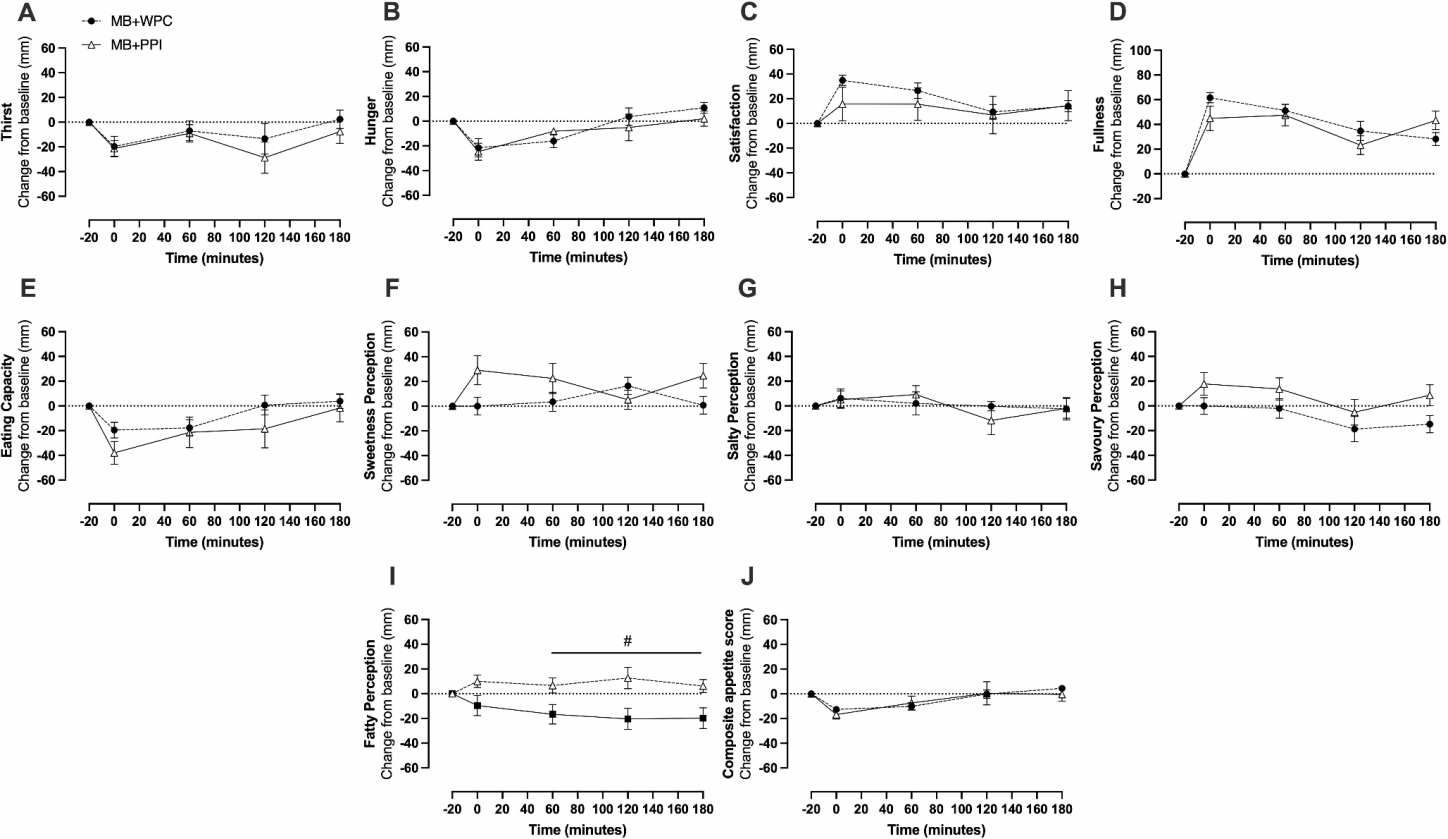
Visual Analogue Scales (VAS) for perceived appetite expressed as change from baseline following mixed breakfast and whey or pea protein supplement. Perceived appetite from VAS scales, presented as change from baseline over 180 min post prandially. Data are presented as mean ± SEM for mixed breakfast with whey protein concentrate (MB + WPC) *n* = 14 and *n* = 11 for mixed breakfast with pea protein isolate (MB + PPI). Thirst (**A**), appetite-related scores (**B–E**), taste perception scores (**F–I**) and composite appetite score (**J**). 2-way ANOVA was run to test for significant difference between groups and timepoints, with baseline value (t=-20) within condition set as the reference point. Significant main effect for time was seen for **A**, **B**, **C**, **D**, **E**, **I** and J (all *P* < 0.05) and significant main effect difference between groups for **I** (*P* < 0.05). Main interaction for **D**, **F** and **I** (all *P* < 0.05). # Denotes significant difference (*P* < 0.05). found between groups over indicated timepoints following significant interaction effect which remained following post hoc analysis

Perceived appetite responses from VAS-derived outcomes showed there was a main effect of time for thirst (*P* = 0.003), hunger (*P* < 0.001), satisfaction (*P* = 0.001), fullness (*P* < 0.001), eating capacity (*P* < 0.001) (Fig. 5A-E) and the composite appetite score (*P* < 0.001) (Fig. 5J), but no main effect of time for any taste preferences (Fig. 5F-I, all *P* > 0.05). There was no significant main effect of group or group and time interaction for any appetite parameter (*P* > 0.05 for all), with the exception of a significant main effect of group (*P* = 0.005) and interaction (Fig. 5I; *P* = 0.011) evident for fatty food perception. Fatty food perception was significantly greater for MB + PPI at 60, 120 and 180 min compared with MB-WPC (*P* < 0.05 for all). There was no significant difference between groups in AUC, time to min or time to peak for any perceived measurement (all *P* > 0.05).

## Discussion

Achieving sufficient protein intake to maximise postprandial muscle anabolism in middle-to-older adults is challenging given the higher muscle anabolic protein dose requirement and/or barriers to consuming such intakes. Pragmatic nutritional strategies including the provision of higher quality protein sources to typically lower protein-containing meals have the potential to augment postprandial MPS stimulation and support longer-term muscle health with advancing age. The present study is the first to characterise the postprandial plasma AA and appetite regulatory response to the ingestion of a lower protein-containing whole food mixed breakfast supplemented with a small bolus of high-quality whey protein concentrate (MB + WPC) or isonitrogenous amount of lower quality pea protein isolate (MB + PPI) in middle-to-older aged adults. Our findings show that MB + WPC and MB + PPI resulted in a comparable postprandial increase in plasma TAA and EAA concentrations, whereas the overall concentration of plasma leucine was greater for MB + WPC than MB + PPI, corroborating the findings of others on isolated pea vs. whey protein-containing drinks [46]. Ingestion of MB + WPC and MB + PPI transiently altered concentrations of plasma glucose, insulin, total GLP-1, and total ghrelin as well as perceived hunger and appetite sensations, with no differences between conditions. Collectively, these findings suggest that the postprandial plasma leucine response following MB + WPC could potentially support a greater muscle anabolic response than MB + PPI in middle-to-older aged adults, without adversely affecting appetite regulation.

Leucine is important in the regulation of intracellular anabolic signalling and as a substrate for MPS [47, 48]. The increase in postprandial plasma leucine concentration has been implicated as a critical driver for MPS stimulation [49]. Furthermore, leucine fortification of lower-dose AA provision has been previously shown to augment postprandial MPS in older adults [50–52]. Based on the divergent leucine profile of WPC and PPI supplements used herein, we hypothesised that postprandial plasma leucinemia would be greater following ingestion of MB + WPC than MB + PPI, indicative of a more favourable condition for postprandial muscle anabolism. In line with our hypothesis, postprandial overall plasma leucine availability was greater with MB + WPC than MB + PPI. This superior leucine response with MB + WPC may augment the MPS response and offer greater net muscle protein balance in middle-to-older aged adults when repeated over multiple feeding opportunities. However, it has recently been suggested that leucine content is subordinate for MPS when combined with whole foods, where additive properties of whole foods may prevail as more beneficial for MPS or lower the amount of leucine needed to maximise anabolism and surpass the ‘leucine threshold’ to trigger MPS [53–55]. Indeed, long-term leucine supplementation (2.5 g/day for 3 months) in healthy older men with a daily protein intake similar to the present study did not augment skeletal muscle mass or strength over placebo [56]. Conversely, the addition of 5 g leucine with low protein meals for 3 days augments muscle protein synthesis rates in older men [57], suggesting high leucine doses may be required to stimulate anabolism. Undoubtedly, the benefit of small increases in leucine (when below the ~ 2.5 g ‘leucine threshold’) alongside whole foods remains to be elucidated. We also acknowledge that the present study was slightly underpowered (1-β = 0.60) using total iAUC leucine availability, where using G*Power (Ver 3.1), *n* = 16 per group is needed to adequately power the study at (1-β = 0.80). Henceforth, it stands to reason that the ~ 29% greater leucine provision with WPC over PPI in the present study, could offer a superior anabolic response over an extended supplementation period.

Indeed, postprandial MPS is regulated by factors other than plasma leucine availability [55]. Specifically, the plasma availability of all EAAs (i.e., not just leucine) are necessary for a robust and sustained postprandial MPS response [58, 59] and longer-term maintenance of muscle remodelling in older adults [56, 60]. In contrast to our hypothesis, there were no significant differences in postprandial EAA availability or peak plasma EAA concentration between MB + WPC and MB + PPI. The absence of differences in EAA concentrations can be reconciled since there were no significant differences in the sum of EAAs between groups. Furthermore, the absence of concentration differences may be masked by ingestion of whole-food MB, slowing the rate of digestion and potentially lowering plasma aminoacidemia [32, 61–64], compared with ingesting supplements in isolated drink form alone [30, 46, 65, 66]. Indeed, McKendry and colleagues [46] recently demonstrated that the co-ingestion of a 25 g whey protein isolate drink with a mixed breakfast meal elicited greater postprandial plasma EAA and leucine availability than ingestion with 25 g of pea protein isolate drink in older adults. Nonetheless, our decision to provide protein supplements in smoothie form alongside the whole food mixed breakfast was taken to represent pragmatic and acceptable means of delivering dietary protein and other important nutrients in a population at increased risk of nutrient deficiencies [67]. Notwithstanding, the comparable postprandial plasma EAA concentrations between groups could indicate a similar capacity for MPS stimulation. It is also worth noting that other non-protein components of the whole food matrix in mixed breakfast meals could influence the postprandial muscle anabolic response [54, 68]. We also acknowledge that the uneven sample size may have impaired our ability to detect a significant difference in postprandial EAA concentrations between groups, the mean of which was ∼27% greater in MB + WPC than MB + PPI. Future studies should seek to understand how the addition of low-dose protein from different sources to meals and snacks in the typical diet of middle-to-older aged adults influences muscle anabolism over an extended free-living period.

Consuming higher protein foods suppresses appetite and hunger more so than lower protein foods [39, 69], a finding that may relate to alterations in appetite-regulating hormones [36, 37]. Whilst the satiating properties of dietary protein hold benefits for weight loss [40, 69] the potential reduction in energy/protein intake at subsequent meals [70] could have a detrimental effect on postprandial muscle anabolism and net muscle protein balance in middle-to-older aged adults, who may already experience age-related impairments in appetite regulation [15]. The satiating effects of protein ingestion may be dictated by the pattern of postprandial AA response [38, 71], particularly the rise in leucine concentrations [72]. In contrast, others have reported no difference in perceived appetite or regulatory hormonal concentrations in response to isolated protein sources that elicit divergent postprandial plasma TAA, EAA and leucine concentrations in younger adults [73, 74]. In line with the present hypothesis, we observed a similar transient suppression of perceived hunger and increased fullness and satiety over a 180 min postprandial period despite the divergent AA content of whey and pea protein supplements and postprandial leucine concentrations following MB + WPC and MB + PPI. Interestingly, hunger and satiety returned to baseline after 180 min, which would be an expected response to accommodate for subsequent protein feeding. The inclusion of an *ad libitum* meal would have confirmed if protein supplementation alongside mixed breakfast does not impair actual eating at the next feeding opportunity, although this was not possible given the present trial was the first meal of a larger controlled dietary intervention. Moreover, the postprandial rise in plasma glucose, plasma insulin, plasma GLP-1 and the decrease in plasma ghrelin did not differ between groups. The only between-group difference observed was a greater desire for fatty food intake in MB + WPC over MB + PPI. It appears that the amount of protein consumed (which was identical between groups in the present study) may be the more important determinant of postprandial appetite regulation [75]. Indeed, the appetite hormones measured in the present study are not exhaustive. Congruent with the present study data, others have demonstrated that appetite markers such as peptide YY, do not differ between high-dose whey and pea protein beverages consumed in isolation [65]. Furthermore, total ghrelin was measured in the present study in only adults with apparently ‘normal’ appetite, whereas acylated ghrelin may be more closely associated with reduced appetite in older adults [15]. Components of the whole food matrix of MB + WPC and MB + PPI may also have influenced appetite regulation, obviating any effect of subtle differences in postprandial aminoacidemia on these parameters. Unfortunately, we were unable to measure how the addition of low-dose whey and pea protein smoothies to the whole food mixed breakfast influenced indices of appetite regulation, which would have required a mixed breakfast-only condition, devoid of supplemental protein. Similarly, providing a higher dose of supplemental protein than 0.13 g·kg BM^− 1^ to achieve a total meal intake of ∼0.4 g·kg BM^1^ to theoretically maximize MPS stimulation in older adults [4] may result in higher satiety and fullness. Addressing these unresolved questions will demonstrate the feasibility of supplementing low protein-containing meals and snacks with additional protein in middle-to-older aged adults.

The present study demonstrates that the addition of a small bolus of whey protein to a lower protein-containing whole-food mixed breakfast (MB + WPC) elicited a greater postprandial plasma leucine response compared with the addition of an isonitrogenous amount of pea protein (MB + PPI) in middle-to-older aged adults. However, postprandial plasma TAA and EAA responses to MP + WPC and MB + PPI were similar. Perceived appetite sensations and appetite regulatory hormone concentrations did not differ between MB + WPC and MB + PPI. Collectively, these findings suggest that MB + WPC is a pragmatic strategy to augment postprandial plasma leucinemia in middle-to-older aged adults, with potential benefits for muscle anabolic stimulation. This finding may have important implications for longer-term muscle health in middle-to-older aged adults, but requires further clarification, particularly in the context of ill-health and hospitalisation where access to, and consumption of, higher protein-containing foods is limited.

## Electronic supplementary material

Below is the link to the electronic supplementary material.

Supplementary Material 1
