# Supplementary material for: Postprandial plasma amino acid and appetite responses to a low protein breakfast supplemented with whey or pea protein in middle-to-older aged adults
Source: Eur J Nutr. 2025 Feb 11;64(2):86. doi: 10.1007/s00394-025-03605-0 (PMC11813961; doi:10.1007/s00394-025-03605-0)
Supplement: Supplementary file 1 — Supplementary Material 1 [file 394_2025_3605_MOESM1_ESM.docx]

## Supplementary Material

**Supplementary Table 1** Relative amino acid contribution from supplemental protein

| Relative protein  (0.13 g×kg BM^-1^) | Whey Protein Concentrate | Pea Protein Isolate | *P* value |
| --- | --- | --- | --- |
| Aspartic Acid | 0.85 ± 0.12 | 0.87 ± 0.24 | 0.814 |
| Serine | 0.42 ± 0.06 | 0.40 ±0.11 | 0.692 |
| Glutamic acid | 1.33 ± 0.19 | 1.21 ± 0.33 | 0.217 |
| Glycine | 0.15 ± 0.02 | 0.30 ± 0.08 | **<0.001*** |
| Histidine | 0.13 ± 0.02 | 0.17 ± 0.05 | **0.017*** |
| Arginine | 0.18 ± 0.03 | 0.59 ± 0.14 | **<0.001*** |
| Threonine | 0.53 ±0.07 | 0.26 ± 0.07 | **<0.001*** |
| Alanine | 0.39 ± 0.05 | 0.31 ±0.08 | **0.009*** |
| Proline | 0.47 ± 0.06 | 0.33 ± 0.09 | **<0.001*** |
| Cystine | 0.19 ± 0.03 | 0.06 ±0.02 | **<0.001*** |
| Tyrosine | 0.19 ± 0.03 | 0.27 ±0.07 | **<0.001*** |
| Valine | 0.36 ±0.05 | 0.29 ± 0.07 | **0.011*** |
| Methionine | 0.19 ± 0.03 | 0.08 ±0.02 | **<0.001*** |
| Lysine | 0.75 ± 0.10 | 0.57 ±0.16 | **0.003** |
| Isoleucine | 0.42 ± 0.06 | 0.25 ±0.07 | **<0.001*** |
| Leucine | 0.75 ± 0.10 | 0.56 ±0.15 | **0.002*** |
| Phenylalanine | 0.23 ± 0.03 | 0.36 ±0.10 | **<0.001*** |
| Tryptophan | 0.13 ± 0.02 | N/A | - |

Amino acid dose (g) provided from supplemental whey protein concentrate (WPC) and pea protein isolate (PPI) as part of smoothie beverages providing 0.13 g×kg BM^-1^ of protein. Data are presented as mean ± SD for low protein mixed breakfast with either WPC (n=13) or PPI (n=9). Independent samples T-test was used to statistically compare relative amino acid content between supplemental protein groups. * Denotes statistical significance between groups (*P*<0.05).


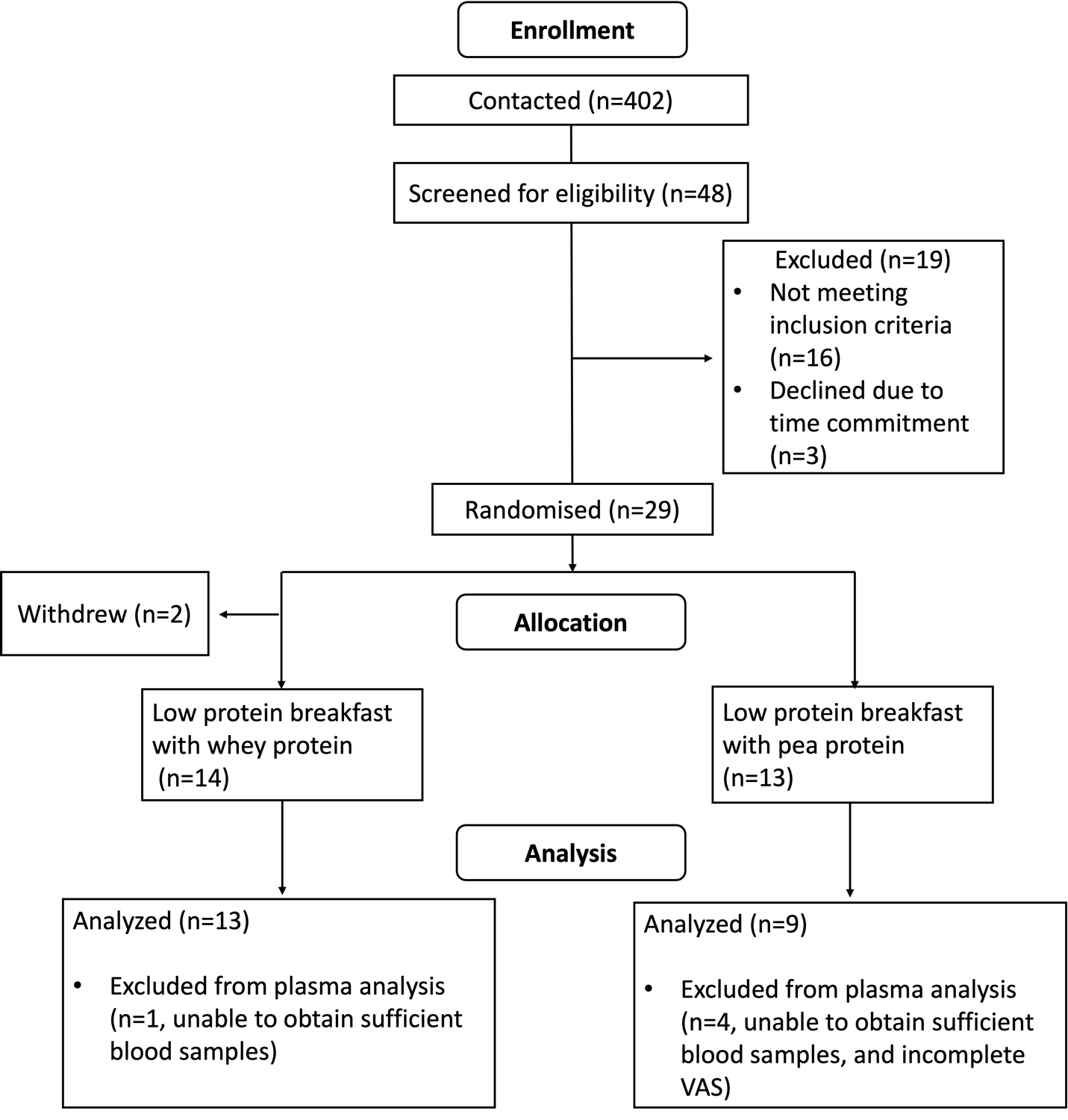


**Supplemental Figure 1**

CONSORT diagram indicating the number of participants enrolled, allocated and then analysed for each outcome measure. Participant drop-out was not related to any aspect of the condition and was due to time commitments of the larger trial.

**Supplementary Figure 2**


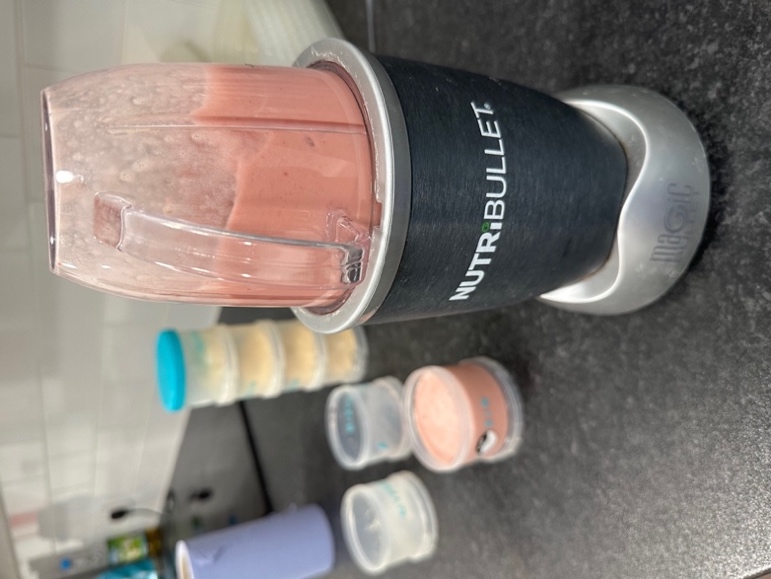


- Add 90ml of whole cranberry juice to blender ahead of other ingredients to minimise residual substance on walls of blender container. To the cranberry juice add 15g of pre chopped frozen banana and 20g of frozen strawberry to provide 35g of whole fruit. Add 0.13 g×kg BM^-1^ of protein powder to the blender container.
- Given that the protein content of the powder differs (80% PPI, 86% for WPC), the absolute amount of powder to be weighed will vary. For a 72kg individual to achieve a serving of 0.13 g⋅kg BM^-1^ the amount of PPI would be 11.7g and WPC would be 10.9g.
- Once the powder is added, secure the lid of the blender container and place in the machine and blitz at full speed for 10-15 seconds until smooth.
- Decant into a container for consumption or into a screw top secure container and refrigerate. If refrigerated, ensure to shake well before consumption and consume within 2 days once blended.


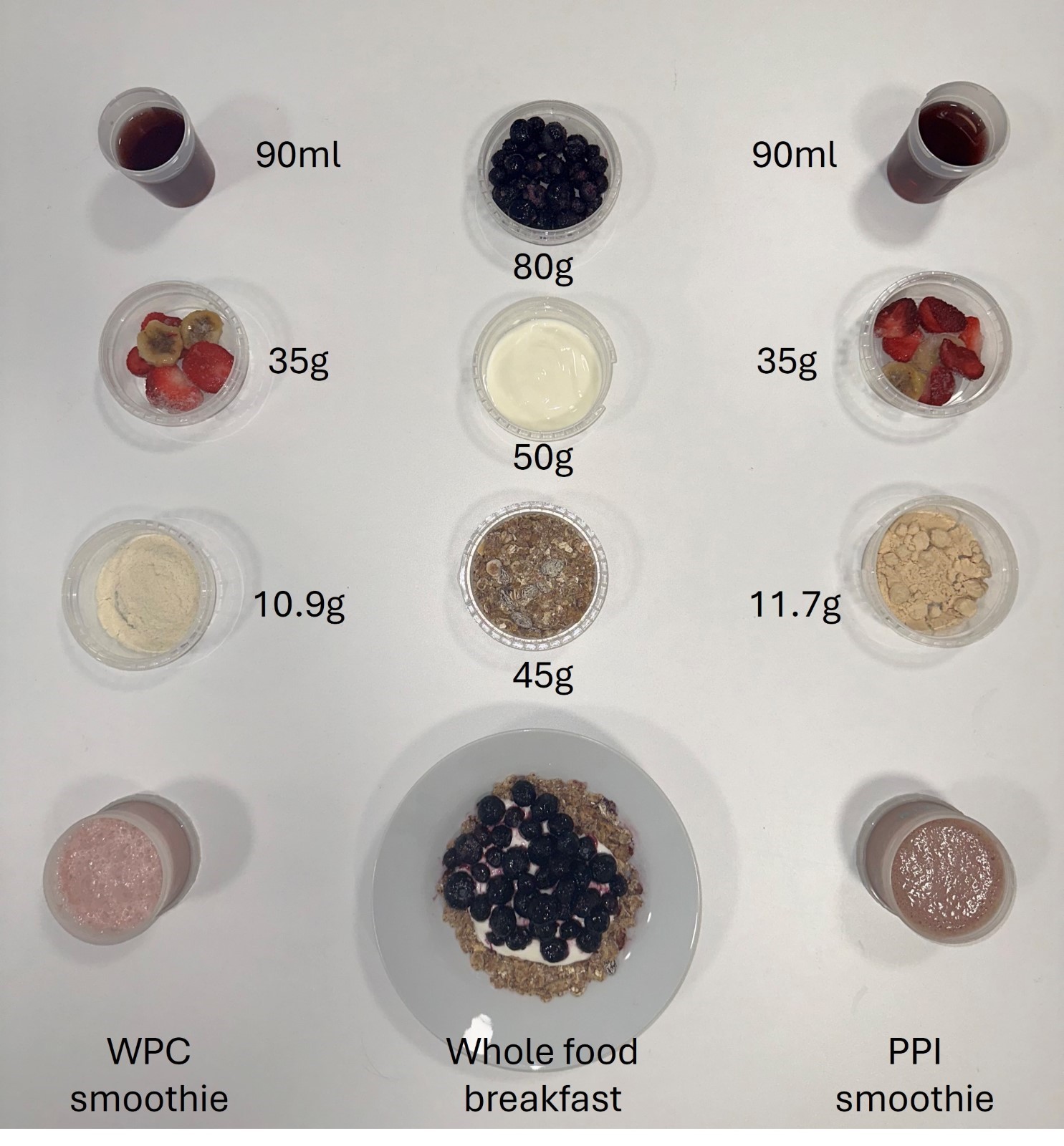


**Supplemental Figure 3**
Image of blended whey protein concentrate (WPC; left) and pea protein isolate (PPI; Right) smoothies with their constituent elements. Both groups consumed the same whole-breakfast test meal (centre) with variations in volume of food according to relative protein intake. Values here are for a 72kg individual where 0.13 g⋅kg BM^-1^ is contributed from one of the supplements and the remaining 0.07 g⋅kg BM^-1^ is from the whole food breakfast.


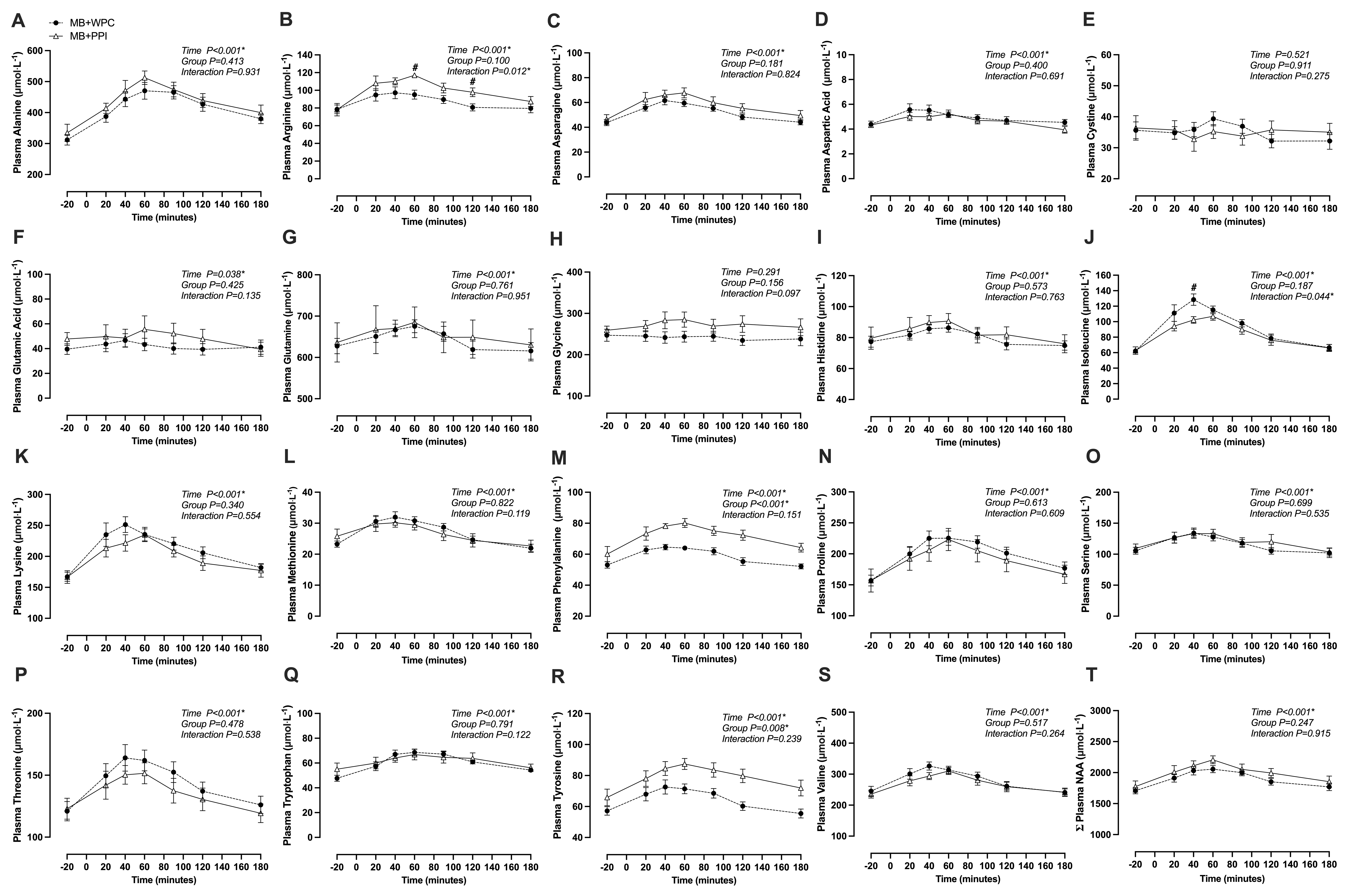


**Supplemental Figure 4** Timecourse for plasma amino acid concentrations.

Postprandial plasma concentrations for individual amino acids (**A-S**) and the sum of all non-essential amino acids (NEAA) (**T**) over 180 min postprandially following ingestion of a mixed breakfast with whey protein concentrate (MB+WPC) or mixed breakfast with pea protein isolate (MB+PPI). Data are presented at mean and SEM with n=13 for MB+WPC and n=9 for MB+PPI. 2-way ANOVA tested for significant main effect difference for time, group and interaction within each figure with baseline value (t=-20) within condition set as the reference value for time differences. *Denotes significant main effect difference from baseline (*P*<0.05) and # for differences between groups (*P*<0.05) designated above each timepoint.

**
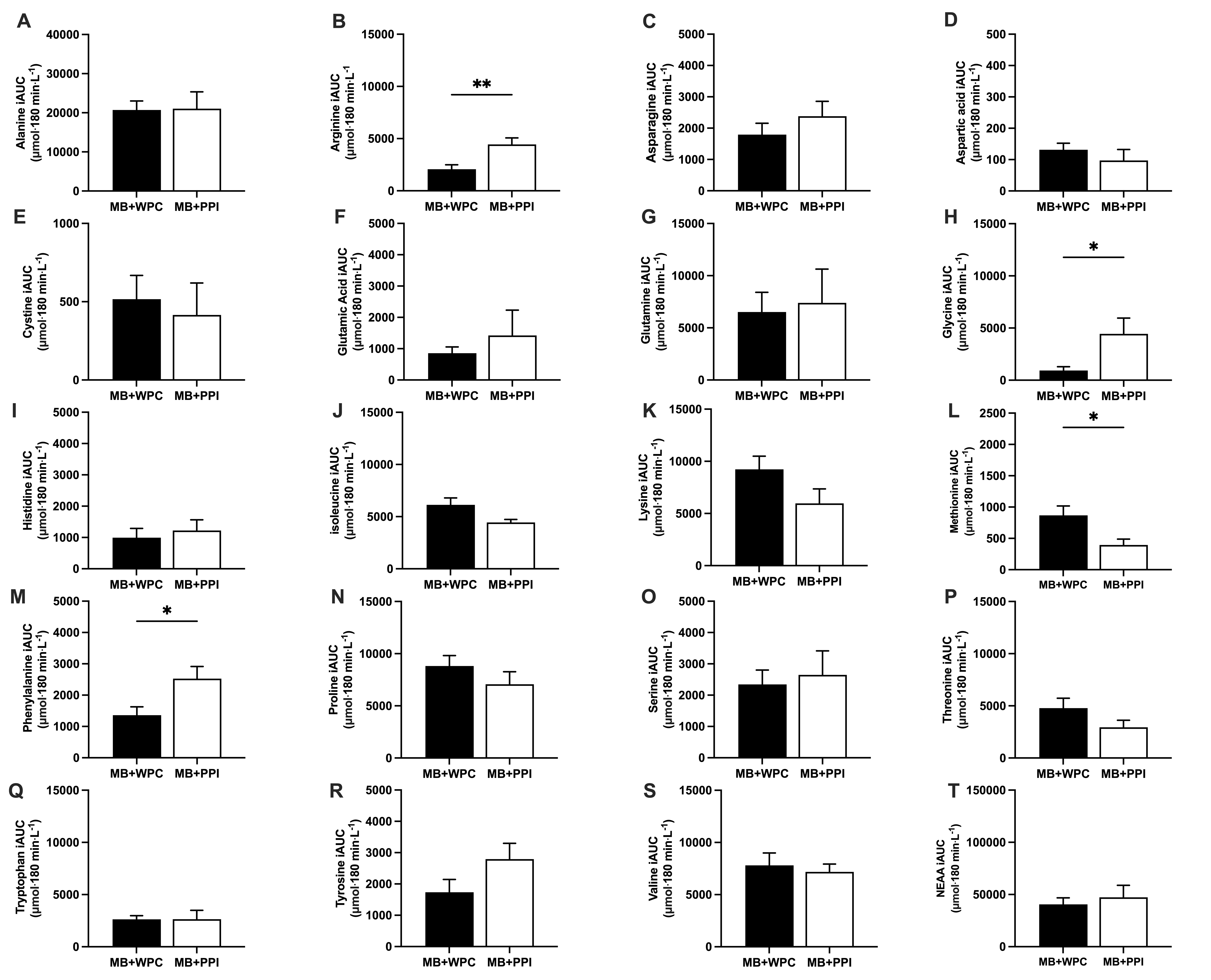
**

**Supplementary Figure 5** Incremental area under the curve for plasma amino acids over the 180 min postprandial period.

Incremental area under the curve (iAUC) over 180 mins for individual amino acids (**A-S**) and sum of non-essential amino acids (NEAA) (**T**) captured in the postprandial period following ingestion a mixed breakfast with whey protein concentrate (MB+WPC) or mixed breakfast with pea protein isolate (MB+PPI). Data are presented at mean and SEM with n=13 for MB+WPC and n=9 for MB+PPI. Independent samples t-test was used to compare between-group differences in iAUC. *Denotes significant difference between groups (**P*<0.05, **P<0.01).

**Supplementary Material 1**

Plasma amino acid concentrations were assessed in collaboration with the Proteomics and Molecular Analysis platform at the Research Institute of the McGill University Health Centre (Montreal, Quebec). Amino acids were extracted from plasma using protein precipitation and derivatized with 6-aminoquinolyl-N-hydroxysuccinimidyl carbamate (AQC; Cayman Chemical, Ann Arbor, Michigan) for analysis using reversed phase ultra performance liquid chromatography mass spectrometry (UPLC-MS). Plasma samples were extracted alongside a calibration curve of amino acids in 0.1N HCl with norvaline as an internal standard (all amino acids and norvaline purchased from Sigma-Aldrich, St. Louis, Missouri, USA). A calibration curve of 5 to 1000 µM was used for all amino acids except cysteine (2.5 to 500 µM). An internal standard working solution (ISWS) containing 25 µM norvaline in 5% 5-sulfosalicylic acid was used to extract plasma and calibration samples. ISWS aliquots (50 µL) were added to sample aliquots (25 µL) in microcentrifuge tubes, vortexed and centrifuged at 15,000 *x g* for 10 mins. Supernatant aliquots (10 µL) were transferred into glass tubes containing 70 µL buffer solution (0.2M sodium borate pH 8.8) along with 20 µL derivatization solution (10mM AQC in acetonitrile), mixed and incubated for 10 min at 55°C. After cooling to room temperature, aliquots (10 µL) were transferred to autosampler vials containing 1000 µL Type-1 water for UPLC-MS analysis. Extracts were analyzed by UPLC-MS using an Agilent 6460 triple quadrupole mass spectrometer coupled with an Agilent 1290 UPLC system (Agilent, Santa Clara, California, USA). Extracts (5 µL) were injected onto an Agilent Eclipse Plus C18 100 x 2.1 mm (1.8 µm) column and chromatographed with a reverse phase gradient at 0.250 mL/min using 0.1% formic acid in water and 0.1% formic acid in acetonitrile. The derivatized amino acids were detected using electrospray positive mode ionization followed by MS/MS fragmentation. Data acquisition was performed using Agilent MassHunter Data Acquisition (version B.04.01) software. Peak area measurements from selected product ions, calibration curve regression analysis and resulting sample quantification were performed using Agilent MassHunter Quantitative Analysis (version B.05.00) software.
